# In Vitro Acid Resistance of Pathogenic *Candida* Species in Simulated Gastric Fluid

**DOI:** 10.1016/j.gastha.2024.100591

**Published:** 2024-11-23

**Authors:** Eri Ikeda, Masaya Yamaguchi, Masayuki Ono, Shigetada Kawabata

**Affiliations:** 1 Department of Microbiology, Graduates School of Dentistry, Osaka University, Osaka, Japan; 2 Center for Infectious Disease Education and Research (CiDER), Osaka University, Osaka, Japan; 3 Bioinformatics Research Unit, Graduates School of Dentistry, Osaka University, Osaka, Japan; 4 Bioinformatics Center, Research Institute for Microbial Diseases, Osaka University, Osaka, Japan

**Keywords:** Artificial Gastric Juice, Candida Albicans, Candida Glabrata, Candida Parapsilosis, Candida Tropicalis

## Abstract

**Background and Aims:**

Although species in the fungal genus *Candida* are often commensal residents of the gastrointestinal (GI) tract, they can also cause high-mortality systemic candidiasis. Most pathogenic *Candida* species are dimorphic fungi that exist predominantly in filamentous forms in the invading tissues. *Candida albicans* is the most prominent pathogen among *Candida* species, but nonalbicans *Candida* species have also emerged as important pathogens. The stomach is the most acidic niche in the GI tract and is maintained at pH 1–2 in healthy individuals. The aim of the present study was to determine whether *Candida* species can survive in gastric fluid and to observe their morphology under varied pH conditions.

**Methods:**

We investigated the in vitro survival of the pathogenic *Candida* species *C. albicans, Candida glabrata, Candida parapsilosis,* and *Candida tropicalis* in simulated gastric fluid.

**Results:**

We first described that a portion of the 4 *Candida* species can survive under highly acidic conditions. Moreover, dimorphic *Candida* species, namely, *C. albicans, C. parapsilosis,* and *C. tropicalis,* exhibited yeast–hyphal transition in simulated gastric fluid with elevated pH. Pathogenic filamentous cells had lower acid resistance than yeast cells.

**Conclusion:**

These findings may illuminate the migration to the lower GI tract by commensal fungi of the oral cavity.

## Graphical abstract

**Figure:**
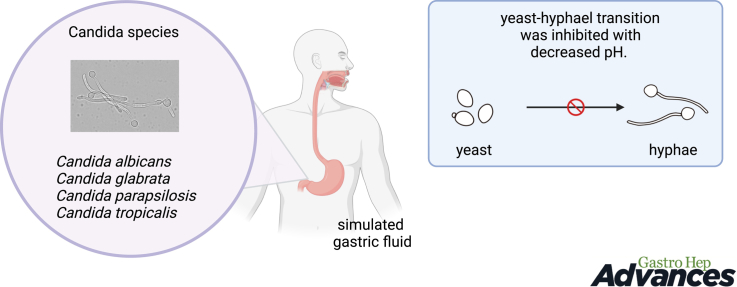

## Introduction

*Candida* species are the most ubiquitous fungi in humans and are both normal resident microorganisms and opportunistic pathogens.^1^ As commensal microorganisms, *Candida* species colonize multiple body sites, such as the skin, vaginal tract, and gastrointestinal (GI) tract, in up to 70% of healthy individuals. *Candida* species are found at multiple sites in the GI tract: in the mouth where digestion begins and in the upper (oesophagus and stomach), middle (small intestine), and lower (colon and rectum) GI tract.^2^^,^^3^ *Candida* become pathogenic and spread abnormally when the host immune defence is weakened, and the infection can range from superficial candidiasis to disseminated disease. The most common manifestation is superficial infection, including oral candidiasis, which affects the mucosa and skin where *Candida* species live as commensals. Invasive candidiasis refers to haematogenous infection, which has up to 40% mortality.^4^ Among *Candida* species, *Candida albicans* is the most common pathogen, accounting for approximately half of the isolates found in epidemiological surveys worldwide. Moreover, in recent years, nonalbicans *Candida* species have emerged as important pathogens causing invasive candidiasis. Specifically, *Candida glabrata, Candida parapsilosis*, and *Candida tropicalis* constitute most of the remaining causes of invasive candidiasis.^5^

The oral cavity contains the second largest human microbiota, comprising more than 500 species of microorganisms.^6^^,^^7^ Healthy humans generally swallow as much as 1.5–2 litres of saliva per day containing extremely high numbers of oral microorganisms.^8^ The stomach, where these microorganisms arrive from the oral cavity, has the lowest potential hydrogen (pH) and is the lowest-biodiversity niche along the GI tract.^2^ Gastric acidity is likely a key factor in the low diversity of the microbiota in the stomach. Intragastric acidity is maintained at approximately pH 1–2 in healthy individuals and temporarily increases to pH 5 during food digestion. Gastric juice is a variable mixture of water, hydrochloric acid, electrolytes, and enzymes, mainly pepsin. Notwithstanding these harsh conditions, the periodontopathic bacterium *Porphyromonas gingivalis* is reportedly resistant to gastric fluid,^9^ and some oral bacteria are found in the GI tract.^10^ Food takes approximately 2–3 hours to pass through the stomach, so microorganisms with short-term acid tolerance may be able to migrate to the lower GI tract.

*C. albicans* is referred to as a polymorphic fungus because it can grow in a yeast, a pseudohyphal, or a hyphal form.^11^ Yeasts live as harmless commensals on mucous membranes. Various environmental cues, such as high temperature, high CO_2_ concentration, and high pH and nutrient deprivation, can trigger yeast-to-hypha morphogenesis. The growth of hyphae is clinically relevant because it is a critical driver of the pathogenesis of symptomatic mucosal infections. *C. albicans* is known to live in a pH range of 2–10,^12^ and acidity suppresses the morphological change to hyphae. Despite these advances in understanding, researchers have not investigated whether *Candida* species can survive passing through highly acidic environments or their morphology under changing pH conditions, especially for pathogenic nonalbicans *Candida* species. Thus, to assess how swallowed *Candida* from the mouth passes through the stomach, we investigated the acid tolerance (particularly towards gastric acid) and morphological changes of 4 pathogenic *Candida* species (*C. albicans, C. glabrata, C. parapsilosis,* and *C. tropicalis*) in vitro.

## Methods

### Candida Strains and Preincubation

*C. albicans* strain SC5314, *C. glabrata* strain ATCC2001, *C. parapsilosis* strain ATCC22019, and *C. tropicalis* strain ATCC750, which are the type strains of their species, were used for this study. *C. albicans* was purchased from ATCC (Manassas, VA, USA). *C. glabrata, C. parapsilosis* and *C. tropicalis* were purchased from RIKEN BRC (Ibaraki, Japan). *Candida* yeast cells were grown in the exponential growth phase on yeast extract–malt extract broth (BD, Franklin Lakes, NJ, USA) at 30 °C. The transformation of *Candida* into filamentous cells was induced on RPMI 1640 medium (Fujifilm Wako Pure Chemical Corp., Osaka, Japan) supplemented with 10% FBS at 37 °C.

### Acid Resistance Assay

Simulated gastric fluid (SGF) was prepared as previously described with minor modifications.^13^ Briefly, 35.6 mM NaCl, 4.48 mM KH_2_PO_4_, 1.0 mM CaCl_2_, and 5.0 mM KCl were added to distilled water, and the solution was autoclaved at 121 °C for 20 min. Pepsin (Fujifilm Wako Pure Chemical Corp.) was added to the stock solution at a final concentration of 4000 U/ml prior to use. The SGF pH was adjusted to pH 1–, 2, 3, 4, or 5 by the addition of HCl before incubation. Yeast extract-peptone-dextrose (YPD) broth (BD, NJ, USA) adjusted to the intended pH was used as a control medium. The pH was measured using a LAQUA F-71 pH meter (Horiba, Ltd, Kyoto, Japan).

### Differences in Candida Cultured at Different pH Values

Preincubated yeast cells were challenged with 1 ml of SGF at pH 1–5 for 4 hours in a 24-well culture dish (Iwaki, Shizuoka, Japan). Cultures were inoculated at a starting optical density of 0.1 at 600 nm (Genesys 50; Thermo Fisher Scientific, MA, USA). Acid survival was evaluated by plating serial dilutions (10^−2^ to 10^−6^) of culture medium after 1, 2, and 4 hours of culture onto yeast extract–malt extract agar plates, incubating for 24 h at 30 °C, and counting the number of colony-forming units. The cultured medium was diluted with phosphate buffered saline, and 20 μl of the diluted medium was used for plating. Untreated control cells were evaluated by the same method. All assays were carried out in sextuplicate.

### Morphological Transformation of Candida

The *Candida* species, except for *C. glabrata,* were photographed under a fluorescence microscope (BZ-X810 and BZ-X710; Keyence, Osaka, Japan) after 4 hours of cultivation with SGF to record their growth form. The numbers of yeast cells and filamentous cells were counted in 15 randomly chosen fields of view. To assess the survival of filamentous cells, filamentous cells induced on RPMI 1640 medium containing 10% FBS at 37 °C overnight were challenged at pH 1 for 4 hours, after which acid survival was quantified as described above.

### Statistics

Welch's t test was used to assess the statistical significance of differences between groups.

## Results

### Candida Species Are Susceptible to SGF at Relatively Low pH

**Figure 1:**
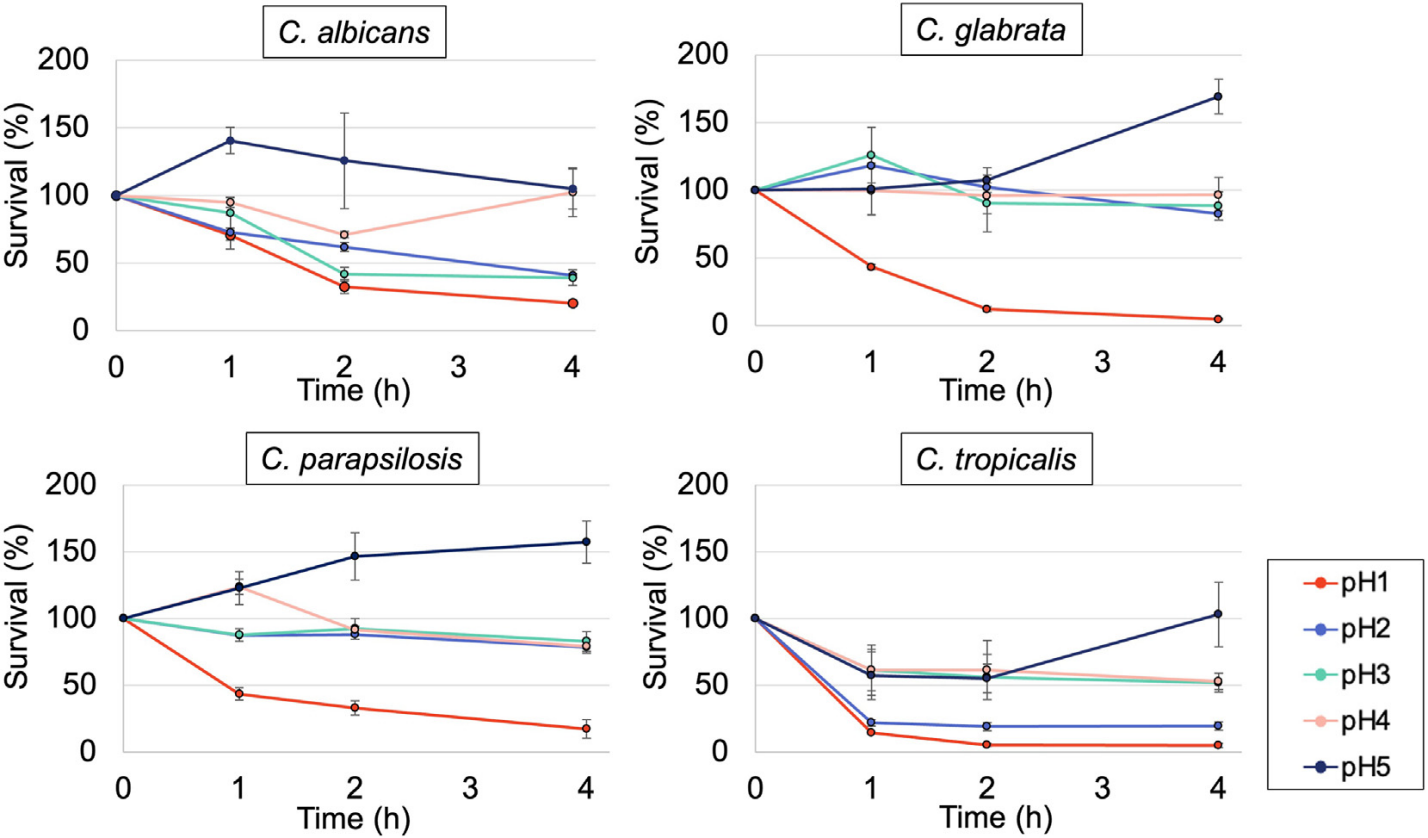
Preincubated cells of 4 *Candida* species, *C. albicans, C. glabrata, C. parapsilosis,* and *C. tropicalis,* were challenged in simulated gastric fluid (SGF) with an initial pH between 1.0 and 5.0 at 37 °C for 4 hours. Survival of the cells was assessed by counting the colonies. Data were presented as mean ± standard deviation (SD). The data in all panels are from 3 independent experiments performed in sextuplicate.

To examine fungal survival in the stomach, preincubated cells of 4 *Candida* species, *C. albicans, C. glabrata, C. parapsilosis,* and *C. tropicalis,* were challenged with SGF adjusted to pH 1–5 for 4 hours. As the acidity increased, the growth of all 4 *Candida* species was suppressed (Figure 1). After 4 hours, the survival rates of *C. albicans, C. glabrata*, *C. parapsilosis*, and *C. tropicalis* in SGF adjusted to pH 1 were 20.3 ± 1.5%, 4.8 ± 1.6%, 17.2 ± 7.0%, and 4.9 ± 2.4%, respectively. The survival rate increased with increasing pH from pH 2–5 and exceeded 100% at pH 5 for all *Candida* species.

### Candida Species Growth Was Attenuated by Low pH and by Gastric Acid Components

**Figure 2:**
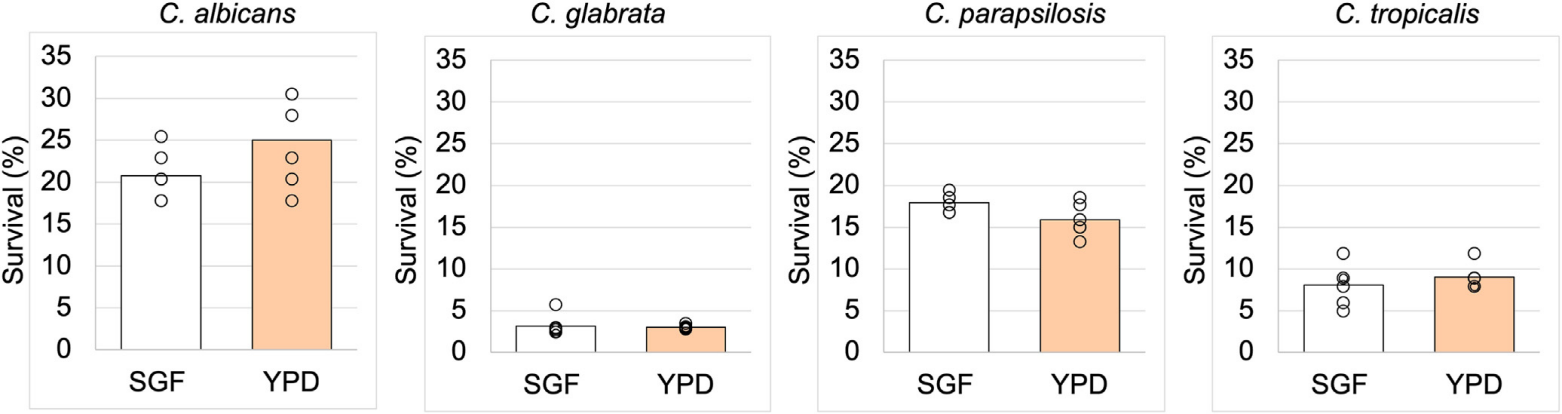
Comparison of *Candida* growth in simulated gastric fluid (SGF) adjusted to pH 1 at 37 °C and yeast extract-peptone-dextrose (YPD) broth adjusted to pH 1 at 30 °C for 4 hours. Survival of the cells was assessed by counting the colonies produced subsequently. Welch's t test was used to assess the statistical significance of differences between groups. Three independent experiments were performed in sextuplicate.

SGF contains the aspartic protease pepsin, which functions optimally at 37 °C and pH 1–2. We evaluated the survival of *Candida* species in SGF without considering the effect of pH. The survival rates of the 4 *Candida* species in SGF adjusted to pH 1 at 37 °C were compared with those in YPD broth adjusted to pH 1 at 30 °C, which are the standard cultivation conditions for *Candida* species except for the acidity. Contrary to our expectations, cultivation in SGF or YPD did not affect the survival rate of *Candida* (Figure 2).

### Morphological Change to Filamentous Forms Was Suppressed at Low pH

**Figure 3:**
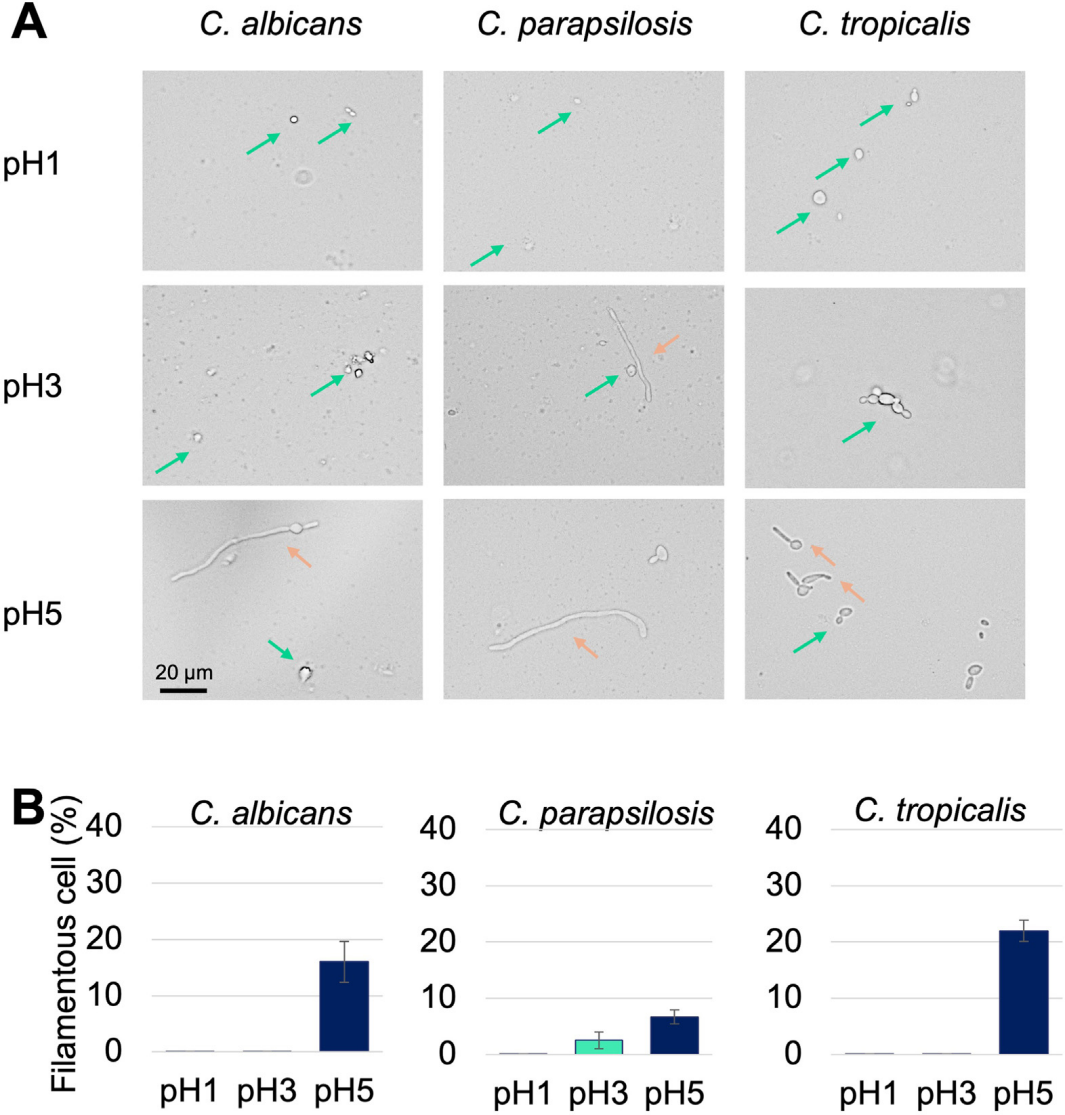
Growth forms of dimorphic *Candida* species cultured in SGF pH 1, 3, and 5 at 37 °C for 4 hours. (A) The growth form was recorded using a camera attached to a microscope. Green arrow indicates yeast cell and orange arrow indicates filamentous cell. (B)The numbers of yeast and filamentous cells were counted in more than 15 fields of view in each group, and the percentages of filamentous cells are indicated. Data were presented as mean ± SD.

Yeast–hyphal transition is a major indicator of pathogenesis and tissue infiltration ability. To better understand the morphological form of *Candida* in the stomach, filamentous changes were observed and counted under a microscope. As *C. glabrata* is a nonhyphae-producing yeast, *C. albicans, C. parapsilosis,* and *C. tropicalis* were used in this experiment. *C. parapsilosis* does not produce true hyphae but can generate pseudohyphae.^14^ In SGF pH 5, filamentous cells were observed for all 3 species, *C. albicans, C. parapsilosis,* and *C. tropicalis,* and the percentages of filamentous cells were 16.0 ± 3.6%, 6.7 ± 1.2%, and 22.0 ± 1.9%, respectively (Figure 3A and B). In contrast, no filamentous cells were found in SGF pH 1. Notably, at pH 3, filamentous cells were observed only for *C. parapsilosis*, and the abundance of these cells was low (2.5 ± 1.4%).

### Filamentous Candida Cells Exhibited Lower Acid Resistance than Yeast Cells

**Figure 4:**
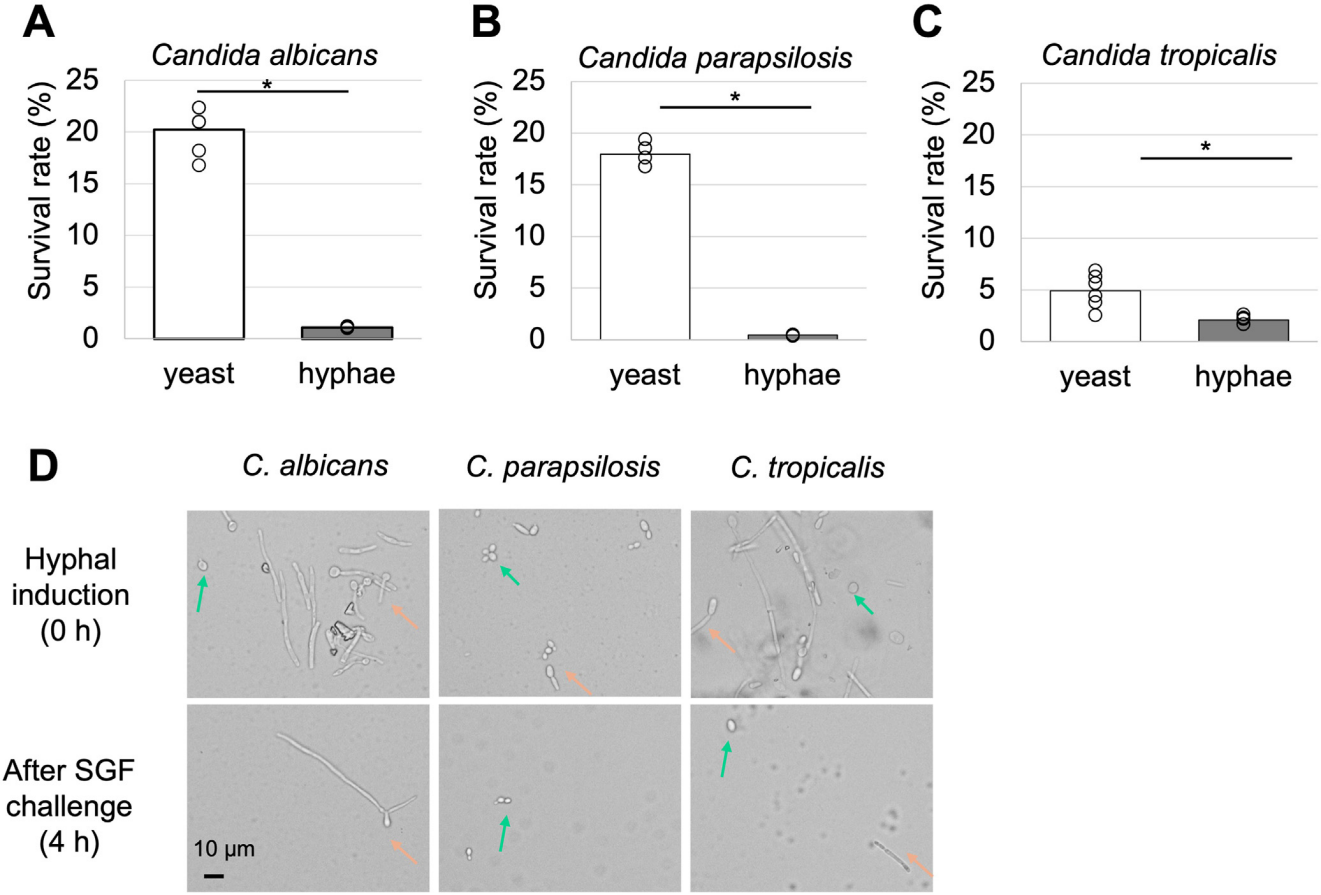
Survival rate was compared between filamentous and yeast cells. Survival rate of *C. albicans* (A), *C. parapsilosis* (B), and *C. tropicalis* (C) in simulated gastric fluid (SGF) with an initial pH of 1.0. (D) The morphology was recorded using a camera attached to a microscope. Green arrow indicates yeast cell and orange arrow indicates filamentous cell. Welch's t-test was used for assessing statistical significance between groups. Three independent experiments were performed in sextuplicate.

Since low-pH SGF inhibited the hyphal/pseudohyphal development of *Candida* species, we examined whether *Candida* filamentous cells transferred from the oral cavity or oesophagus could survive in the stomach. Therefore, we next tested the survival of *Candida* filamentous cells in SFG with high acidity. *C. albicans, C. parapsilosis,* and *C. tropicalis* hyphal cells were induced by preincubation overnight in RPMI 1640 medium supplemented with 10% FBS. In contrast to yeast cells, only 1.1 ± 0.1%, 0.5 ± 0.04%, and 2.1 ± 0.3% of filamentous cells survived after 4 hours of incubation in SGF pH 1 (Figure 4A–C). The morphology of *C. albicans, C. parapsilosis* and *C. tropicalis* after SGF challenges were mixture of both yeast and filamentous cells (Figure 4D). The survival rates of filamentous cells in SGF pH 1 were significantly lower than those of yeast cells for all 3 species, indicating that highly acidic SGF impairs not only the morphological change to filamentous cells but also filamentous cell survival.

## Discussion

The stomach is the most acidic organ in the GI tract, and it is believed that gastric hydrochloric acid kills most microorganisms that invade this organ.^15^ However, whether *Candida* can pass through the stomach is poorly understood. Herein, we showed that some proportion of pathogenic *Candida* species, namely, *C. albicans, C. glabrata, C. parapsilosis,* and *C. tropicalis*, can survive for hours in SGF, mainly as yeast. We further showed that increasing the pH increased the survival rate of all 4 *Candida* species and promoted yeast–hyphal transition in dimorpahic *Candida* species.

The overgrowth of *Candida* species in several areas of the GI tract has been investigated. The oral cavity, the uppermost part of the GI tract, is one of the main reservoirs of *Candida* species. The overgrowth accumulation of *Candida* in the mouth, called oral candidiasis, often develops in immunocompromised hosts including elderly people or patients with HIV. Swollen *Candida* cells of oral candidiasis patients are about 1∼2 × 10^6^ colony-forming unit per day.^16^ These overgrown fungi can be swallowed with saliva and pass through the throat and oesophagus to enter the stomach.^17^^,^^18^ Gastric candidiasis is predominantly observed in patients with peptic ulcer disease or gastric carcinogenesis.^19^^,^^20^ PPIs and histamine H2-receptor antagonists, the main prescriptions for peptic ulcer treatment, decrease the amount of acid produced by the stomach to reduce irritation to the stomach lining and allow ulcers to heal.^21^ In addition to suppressing gastric acid production, the administration of PPIs or H2-receptor antagonists increases the gastric pH in most patients from 2.0 to 6.0, which facilitates the colonization of the stomach by *Candida* species.^22^^,^^23^ In a cohort study, H2RA therapy induced *Candida* overgrowth in the gastric fluid of peptic ulcer patients, and fungal colonization in the stomach was associated with the inhibition of disease healing.^24^ Furthermore, PPI users exhibited fungal dysbiosis and a higher abundance of *Candida* species in the gut.^25^ In agreement with the finding, hypochlorhydria resulting from PPI use was reported as the most common risk factor associated with *Candida* oesophagitis and intestinal fungal overgrowth.^26^, ^27^, ^28^ The gut contains the largest microbiome in the human body, and the disruption of the microbiome, called dysbiosis, is closely connected to pathological states such as inflammation. For example, inflammatory bowel disease (IBD) is a chronic, life-long condition that can be treated but not cured.^29^ Several factors are implicated in IBD development, including gut dysbiosis,^30^ reduced integrity of the intestinal barrier, and dysfunction. It has been reported that *Candida* species, especially *C. albicans, C. glabrata,* and *C. tropicalis*, were present in higher abundance in the gut microbiome of IBD patients, suggesting the association of these *Candida* species with IBD.^31^ The 4 *Candida* species used in the present study were found to be present as commensal microorganisms in both the oral cavity and the gut of healthy individuals.^32^^,^^33^ To our knowledge, the hypothesis that *Candida* colonization of other parts of the GI tract originates from the oral cavity and has not yet been confirmed. However, our observations in the present study and the results of previously reported epidemiological studies support this idea. *Candida* cells that are swallowed from the mouth and further survive the stomach acid may not only settle in the gut but also cause gut dysbiosis, and thus the onset or development of diseases related to gut dysbiosis.

The Rim101 signal transduction pathway governs pH responses and differentiation and senses and responds to neutral-alkaline environments.^34^, ^35^, ^36^ An acidic pH promotes yeast morphology by impairing the Rim101-dependent profilamentation signalling pathway in *C. albicans*.^37^ A system of response correlated to the population density of microorganisms is known as quorum sensing. A quorum sensing molecule of *C. albicans*, farnesol also inhibits hyphal initiation as the cell concentration increases.^38^ Growth in acidic environments decreases the expression of the cell wall chitinase Cht2, and thus increases chitin and β-glucan exposure at the cell wall periphery.^39^ Immunostimulatory β-glucan was found to accelerate the recognition of *C. albicans* by innate immune cells in a mouse infection model. In addition, GI colonization of orally administrated *Candida* species (*C. albicans, C. glabrata, C. parapsilosis,* and *C. tropicalis*) was observed previously.^40^, ^41^, ^42^, ^43^, ^44^ However, the pH of the rodent stomach is generally considered to be 3–5, which is less acidic than that of humans,^45^ which hinders in vivo studies to determine the behaviour of *Candida* species under highly acidic conditions. Thus, our in vitro study demonstrated the potential limitations in predicting the behaviour of *Candida* in the human GI tract.

In conclusion, we first demonstrated that 4 *Candida* species responsible for invasive candidiasis, namely, *C. albicans, C. glabrata, C. parapsilosis,* and *C. tropicalis*, endure gastric acid challenges in vitro and the survival rate varied among species. Filamentous cell survival and the morphological transition from yeast to filamentous cells were impaired in highly acidic SGF, suggesting that pathogenic filamentous cells hardly endure acidity in healthy individuals. That said, the presence of filamentous cells increased with increasing simulated intragastric pH. Our results showed the possibility that *Candida* species could pass through the stomach, especially in GI disease patients who receive medications that alter the intragastric pH.
